# ti-scMR: trajectory-inference-based dynamic single-cell Mendelian randomization identifies causal genes underlying phenotypic differences

**DOI:** 10.1093/nargab/lqaf082

**Published:** 2025-07-04

**Authors:** Jianle Sun, Qun Dong, Jialu Wei, Yan Gao, Zhangsheng Yu, Xiaowen Hu, Yue Zhang

**Affiliations:** Department of Bioinformatics and Biostatistics, School of Life Sciences and Biotechnology, Shanghai Jiao Tong University, Shanghai, 200240, China; Department of Philosophy, Carnegie Mellon University, Pittsburgh, PA, 15213, United States; Department of Bioinformatics and Biostatistics, School of Life Sciences and Biotechnology, Shanghai Jiao Tong University, Shanghai, 200240, China; Department of Bioinformatics and Biostatistics, School of Life Sciences and Biotechnology, Shanghai Jiao Tong University, Shanghai, 200240, China; Bio-X Institutes, Key Laboratory for the Genetics of Developmental and Neuropsychiatric Disorders (Ministry of Education), Shanghai Jiao Tong University, Shanghai, 200030, China; Department of Bioinformatics and Biostatistics, School of Life Sciences and Biotechnology, Shanghai Jiao Tong University, Shanghai, 200240, China; SJTU–Yale Joint Center for Biostatistics and Data Science Organization, Translational Science Institute, Shanghai Jiao Tong University, Shanghai, 200240, China; Clinical Research Institute, Shanghai Jiao Tong University School of Medicine, Shanghai, 200025, China; Center for Biomedical Data Science, Translational Science Institute, Shanghai Jiao Tong University, Shanghai, 200240, China; Bio-X Institutes, Key Laboratory for the Genetics of Developmental and Neuropsychiatric Disorders (Ministry of Education), Shanghai Jiao Tong University, Shanghai, 200030, China; Department of Bioinformatics and Biostatistics, School of Life Sciences and Biotechnology, Shanghai Jiao Tong University, Shanghai, 200240, China; SJTU–Yale Joint Center for Biostatistics and Data Science Organization, Translational Science Institute, Shanghai Jiao Tong University, Shanghai, 200240, China; Center for Biomedical Data Science, Translational Science Institute, Shanghai Jiao Tong University, Shanghai, 200240, China; Soong Ching Ling Institute of Maternity & Child Health, Shanghai, 200230, China

## Abstract

The selective expression of genes is the basis of cellular and individual phenotypic differences, serving as a mediator in the causal pathways from genotypes to phenotypes. Single-cell differential expression analysis identifies distinct transcriptomic landscapes, but fails to establish causal relationships due to the presence of confounders. On the other hand, causal inference methods in population genetics such as Mendelian randomization often overlook the heterogeneity among cells and dynamic changes along trajectory. To address these limitations, we propose the trajectory-inference-based dynamic single-cell Mendelian randomization (ti-scMR), integrating population genomes and single-cell transcriptomes to explore transcriptional features causally linked to cellular and individual phenotypes. ti-scMR leverages trajectory inference and functional principal component analysis to capture the temporal cumulative effects of gene expression, select genetic instrumental variables through single-cell expression quantitative trait locus (eQTL) mapping, and employ transcriptome-level Mendelian randomization to prioritize causal genes for cellular and individual phenotypes, specifically those that are related through affecting cellular development. We demonstrate the superiority of ti-scMR in identifying causal genes through simulations. With application in two real single-cell datasets, we discover potential causal genes on immune cell differentiation and related disease. The integration of single-cell trajectory inference, eQTL, and Mendelian randomization will make ti-scMR a powerful tool for elucidating the causal mechanisms underlying complex traits.

## Introduction

The selective expression of genes plays a crucial role in generating cellular and individual phenotypic differences, acting as a mediator in the causal mechanism linking genotypes to phenotypes [1]. Advancements in single-cell RNA sequencing (scRNA-seq) promote the identification of numerous distinct transcriptional patterns in different cellular contexts through the application of differential expression analysis (DE). However, understanding the causal relationship between transcriptional differences and phenotypic outcomes remains challenging due to the presence of unobserved confounding.

Meanwhile, population genetics methods, such as colocalization [2], Mendelian randomization (MR) [3], and transcriptome-wide association studies (TWAS) [4], aim to unravel causal relationships. These methods leverage genetic variations as “anchors” [5] to integrate signals from various genetic association studies, including genome-wide association studies (GWAS) and expression quantitative trait locus (eQTL) mapping. Specifically, MR employs genetic variations (typically single nucleotide polymorphisms, SNPs) as instrumental variables (IVs) to elucidate causal links [6]. By specifying gene expression levels as exposures, MR can be performed at the transcriptomic level to identify causal genes that influence individual and cellular phenotypes [7].

Conventional MR approaches are limited by their reliance on large-scale genetic association studies at the population level, failing to capture heterogeneity among different cells or dynamic changes in gene expression during cellular development. The increasing availability of population-level scRNA-seq data has enabled the integration of single-cell genomics with population genetics through single-cell eQTL (sc-eQTL) mapping [8, 9]. Using cell-specific eQTLs as IVs, we can naturally extend MR to the realm of single-cell genomics. Although the prevailing strategy, which aggregates cells into “pseudo-bulk” samples [10], helps reduce noise, it comes with trade-offs in terms of statistical power and the loss of heterogeneity information. On the other hand, an alternative strategy, which treats different cells from the same individual as independent samples, fails to account for their genetic similarity and temporal links throughout cell development. Since cells within an individual actually exist at different developmental stages, trajectory inference (TI) arranges cells within an individual along an inferred temporal lineage with pseudotime points, enabling the modeling of dynamic transcriptomic patterns.

In this article, we propose a method called trajectory-inference-based dynamic single-cell MR (ti-scMR). Our approach leverages TI and sparse functional principal component analysis (FPCA) techniques to capture the cumulative offsets of dynamic gene expression. Incorporating genetic instruments identified by sc-eQTL mapping, we can extend time-cumulative MR to the transcriptome level, revealing putative causal genes linked to phenotypic differences. Through the applications of ti-scMR on two real single-cell datasets, we demonstrate its exciting potential in exploring causal genes that influence individual phenotypes (such as diseases) as well as generalizing to cellular phenotypes (such as cell types). We believe this methodological advancement in combining single-cell genomics and statistical genetics represents a significant step forward in elucidating the causal mechanisms driving complex traits and phenotypes.

## Materials and methods

### Model overview

**Figure 1.**
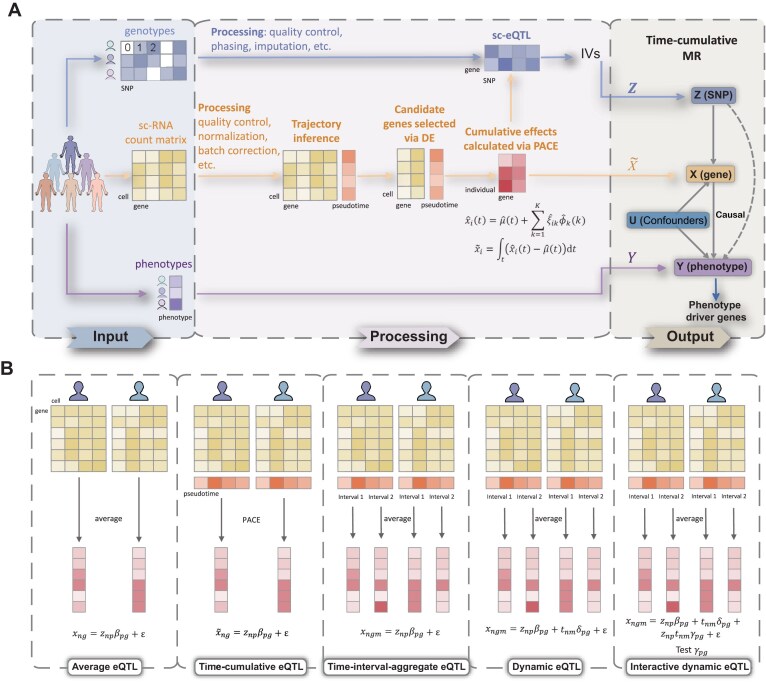
Model overview. (**A**) ti-scMR integrates population genomes and single-cell transcriptomes to identify genes causally linked to phenotypes. We capture the dynamic information of gene expression through TI and FCPA, select genetic IVs via sc-eQTL mapping, and identify causal genes with single-cell transcriptome-level MR. (**B**) Different strategies of sc-eQTL mapping. Average eQTL (avg_eqtl) uses the average expressions of a certain type of cell in each individual as a new sample. Time-cumulative eQTL (cum_eqtl) takes each participant’s cumulative expression effect calculated by PACE as a new sample. Time-interval-aggregate eQTL (agg_eqtl) averages the expression of cells within different time intervals to create a “pseudo-individual” as a new sample. Dynamic eQTL (dyn_eqtl) incorporates each interval’s pseudotimes as covariates into agg_eqtl. Interactive dynamic eQTL (int_eqtl) tests the interactive effects between expression levels and pseudotimes in dyn_eqtl.

ti-scMR integrated population genomes and single-cell transcriptomes to identify genes whose expressions are causally related to changes in cellular and individual phenotypes (Fig. 1A). Based on the inferred trajectory, we modeled the temporal change in the reconstructed cell trajectory using the principal analysis by conditional estimation (PACE), an algorithm of FPCA on sparse and irregular longitudinal data [11], and then estimated the time-cumulative offsets of gene *g*.

Since the effects of gene expression on phenotypes are usually cumulative rather than instantaneous, we specified the cumulative expression offsets estimated from PACE as exposures, and employed a time-varying MR framework [12] to discover causal genes that influence the phenotype. Using SNPs identified through sc-eQTL mapping as genetic instruments, we can employ one-sample MR estimators, such as two-stage least squares (2SLS) and two-stage residual inclusion (2SRI) [13], to test whether the expression of each gene has a significant effect on the phenotype. Lasso penalization was adopted to control horizontal pleiotropy [14].

In summary, ti-scMR uses the time-cumulative eQTL (cum_eqtl) in IV selection, and pace-estimated cumulative effects as exposures with lasso-regularized 2SLS or 2SRI estimators (pace_linear_lasso or pace_logit_lasso) in MR test. Finally, ti-scMR prioritizes genes based on the *P*-values from MR tests for each gene, identifying a set of potential causal genes that affect individual-level phenotypes (e.g. diseases) or molecular traits (e.g. cell differentiation outcomes).

### Preprocessing and TI of single-cell transcriptome

Single-cell transcriptomes are typically characterized by various sources of noise, such as sequencing depth, batch effects, and dropouts. The sparsity and non-Gaussianity make it unsuitable for ordinal PACE algorithm. We applied the Seurat standard workflow [15] for preprocessing the single-cell transcriptomic data, including quality control, log normalization, scaling, and batch effect correction using Harmony [16, 17]. We performed TI using slingshot [18].

Additionally, we employed a “time-interval aggregation” technique to mitigate measurement errors, which means dividing the inferred trajectory into fewer intervals (by rounding the inferred pseudotime points) and using the midpoint of each interval as a representative for subsequent analyses. Within each interval, cells were averaged for each individual. We utilized the PACE method to estimate the cumulative expressive effects of each gene for each individual, followed by a subsequent MR analysis.

### Time-cumulative offsets of gene expression

The effects of gene expression are usually cumulative rather than instantaneous. Therefore, we leveraged an epidemiological model that characterizes cumulative offsets of exposure factors before MR [12] to help identify genes in single-cell transcriptomics that have causal effects on cellular and individual phenotypes.

For a time series *x*_*i*_(*t*) of exposure *X*, we can perform FPCA,


(1) \begin{eqnarray*}
x_i(t)={\mu} (t)+\sum _{k=1}^{\infty}\xi _{ik}\phi _k(t),
\end{eqnarray*}

where $\mu (t) = \mathbb {E}[x(t)]$ is the mean function, and the functional principal components (PCs) come from the spectral decomposition of the covariance function $R(s,t)=\mathrm{cov}(x(s),x(t))=\sum _{k=1}^{\infty }\lambda _k\phi _k(s)\phi _k(t)$, with the coefficients $\xi _{ik}=\int _t\left(x_i(t)-\mu (t)\right)\phi (t)\mathop {}\!\mathrm{d}t$. Longitudinal data are usually sparse and irregular, and we use the PACE algorithm to estimate the PCs [11]. For the *j*th observation of the *i*th individual, we have


(2) \begin{eqnarray*}
x_{ij}=\mu (t_{ij})+\sum _{k=1}^{\infty }\xi _{ik}\phi _k(t_{ij})+\varepsilon _{ij},
\end{eqnarray*}

where the coefficients $\hat{\xi }_{ik}=\mathbb {E}[\xi _{ik}|\boldsymbol{X}_i]=\lambda _k\boldsymbol{\phi }_{ik}^T\boldsymbol{\Sigma }^{-1}_{\boldsymbol{X}_i}(\boldsymbol{X}_i-\boldsymbol{\mu }_i)$. The complete trajectory can be restored using the first *K* PCs


(3) \begin{eqnarray*}
\hat{x}_i(t)=\hat{\mu }(t)+\sum _{k=1}^K\hat{\xi }_{ik}\hat{\phi }_k(t),
\end{eqnarray*}

and we can estimate the time-cumulative effect of exposure,


(4) \begin{eqnarray*}
\tilde{x}_i=\int _t\left(\hat{x}_{i}(t)-\hat{\mu }(t)\right)\mathop {}\!\mathrm{d}t.
\end{eqnarray*}

The gene expression data are normalized and scaled before PACE to meet the requirement of Gaussianity. We implemented the PACE algorithm with R package fdapace, determined the value *K* based on the fraction of variance explained by the PCs (FVE) exceeding 99%, and calculated numeric integral with R package pracma.

### sc-eQTL mapping with temporal information

To demonstrate the benefits of incorporating temporal information inferred by TI, we compared the following different sc-eQTL approaches (Fig. 1B).

- Average eQTL (avg_eqtl): using the average expression of a certain type of cell in each individual as a new sample (pseudo-individual) and applying the ordinary eQTL methods for bulk data [19].
- Time-cumulative eQTL (cum_eqtl): taking each participant’s cumulative expression effect calculated by PACE as a new sample, and then using the bulk eQTL methods.
- Time-interval-aggregate eQTL (agg_eqtl): dividing the trajectory into fewer intervals, averaging the cell expression of each individual in each interval to create a “pseudo-individual” as a new sample, and then using the bulk eQTL methods.
- Dynamic eQTL (dyn_eqtl): as time-interval-aggregate eQTL, using the interval-aggregate “pseudo-individuals” as the samples, but with corresponding pseudotime (midpoint of each interval) as covariates, and then using the bulk eQTL methods.
- Interactive dynamic eQTL (int_eqtl): like the dynamic eQTL, using the interval-aggregate “pseudo-individuals” as the samples and incorporating pseudotimes as covariates, but testing the significance of the interaction effect between genotypes and pseudotimes.

eQTL analysis was implemented with the R package MatrixEQTL. For significant eQTL loci [false discovery rate (FDR) < 0.05], the linkage disequilibrium (LD) stepwise clumping was used to remove SNPs in LD (*r*^2^ > 0.1).

### Time-cumulative effect MR

Treating the time-cumulative offset $\tilde{x}_i$ as the exposure, we can perform a one-sample MR analysis using 2SLS,


(5) \begin{eqnarray*}
\begin{aligned} \tilde{x}_i&=\alpha _0+\boldsymbol{\alpha }^T\boldsymbol{Z}+\varepsilon _x,\\ y_i&=\beta _0+\beta \hat{\tilde{x}}_i+\varepsilon _y, \end{aligned}
\end{eqnarray*}

where $\boldsymbol{Z}$ is SNPs selected as IVs.

For the purpose of hypothesis testing, binary outcomes can be treated as continuous variables and directly analyzed using 2SLS, while IV estimators for binary outcomes have also been proposed, including two-stage predictor substitution (2SPS) and 2SRI [13, 20, 21]. In 2SPS, similar to 2SLS, the predicted value of $\tilde{x}_i$ is included in the second-stage regression, but binary outcomes are modeled using log-linear or logistic models. On the other hand, 2SRI incorporates the residuals of the first-stage regression into the second-stage nonlinear regression model. Previous studies have shown that 2SRI can mitigate bias in certain cases [13, 21], and we integrate 2SRI estimator into our model,


(6) \begin{eqnarray*}
\begin{aligned} \tilde{x}_i&=\alpha _0+\boldsymbol{\alpha }^T\boldsymbol{Z}+e_x,\\ \frac{\mathbb {E}[y_i]}{1-\mathbb {E}[y_i]}&=\beta _0+\beta \tilde{x}_i+\beta _e\hat{e}_x. \end{aligned}
\end{eqnarray*}

To test the significance of *β*, we employed Wald test with robust standard errors calculated by Terza method [22].

To control the influence of potential horizontal pleiotropy, we explicitly incorporated pleiotropic terms into the 2SLS or 2SRI models with Lasso regularization [14]. The optimal penalty coefficient was determined by 10-fold cross-validation. We then obtained the causal effect estimates on the complete dataset and derived the bootstrapping percentile confidence intervals using the optimal penalty coefficient, implemented with the R package boot and boot.pval.

### Simulation of population-level dynamic single-cell transcriptomes

To simulate dynamic single-cell transcriptomes at the population level, we considered different factors contributing to heterogeneity among cells of the same type, including dynamic changes along the trajectories, the eQTL effects within different individuals, and sequencing noise. We first implemented cell sampling along the trajectory based on the single-cell simulation program PROSSTT [23], and incorporated the eQTL effects among different individuals following SplatPop [24].

The genotype matrix $\boldsymbol Z_{N\times P}$ of *P* SNPs and *N* individuals can be simulated using the binomial distribution $\mathcal {B}(2,\pi )$, with effect allele frequencies *π* from the uniform distribution. We also directly sampled real genotypes from European participants in the UK BioBank to mimic genuine LD structures in genomes.

For each individual *n*, we generated the expression levels of *G* genes for *M*_*n*_ cells at different positions on the trajectory based on PROSSTT [23]. Gene expressions are controlled by expression programs that are far less than the number of genes. Using *K* expression programs obtained based on random walk simulation as the basis function *w*_*k*_(*t*), we can calculate the relative mean expression of each gene at different pseudotime points:


(7) \begin{eqnarray*}
\mu _g^{(r)}(t)=\sum _{k=1}^Kw_k(t)h_{kg},
\end{eqnarray*}

where *h*_*kg*_ obeys the gamma distribution Γ(1/*K*, 1). The scaling value of each gene ϕ_*g*_ is sampled from lognormal distribution $\log (\phi _g)\sim \mathcal {N}(0.8,1)$, and we have the absolute mean expression of gene *g* at pseudotime *t*,


(8) \begin{eqnarray*}
\mu _g(t)=\phi _g\mu ^{(r)}_g(t).
\end{eqnarray*}

We considered the eQTL (SNP–gene) effects of different genotypes in a population. If there are *P*_*g*_ SNPs affecting the expressions of gene *g* and the effect size of the *p*th SNP at pseudotime *t* is δ_*pg*_(*t*), the total effect is


(9) \begin{eqnarray*}
\lambda _{ng}(t)\sim \mathcal {N}\left(\sum _{p=1}^{P_g}Z_{np}\delta _{pg}(t),\sigma ^2\right),
\end{eqnarray*}

where *Z*_*np*_ represents the genotype of the *p*th SNP of individual *n*, and δ_*pg*_(*t*) are specified in different forms:

- constant effect: δ_*pg*_(*t*) is a constant value δ_*pg*_, which comes from $\Gamma (3.6 ,{1}/{12})$;
- time-varying effect: δ_*pg*_(*t*) is a sine function $\sqrt{2}\delta _{pg}|\sin (t)|$ that changes with pseudotime *t*, where δ_*pg*_ is generated from $\Gamma (3.6,{1}/{12})$.

Consequently, the average absolute expression level of the gene *g* for the *n*th individual at pseudotime *t* is [24]


(10) \begin{eqnarray*}
\mu _{ng}(t)=\mu _g(t)+\lambda _{ng}(t)\mu _g(t).
\end{eqnarray*}

Using the average expression, we then simulated observed single-cell transcriptomes along the trajectory with sequencing noise. For the *m*th cell of individual *n* at pseudotime *t*, we have


(11) \begin{eqnarray*}
x_{nmg}=\text{NegBin}\left(s_{nm}\mu _{ng}(t),\sigma _{nmg}^2(t)\right),
\end{eqnarray*}

where the variance $\sigma _{nmg}^2(t)=\alpha _g\left(s_{nm}\mu _{ng}(t)\right)^2+\beta _g\left(s_{nm}\mu _{ng}(t)\right)$. We sampled sequencing depth *s*_*nm*_ from log-normal distribution $\log (s_{nm})\sim \mathcal {N}(\log (1),\log (0.7)^2)$, α_*g*_ from $\log (\alpha _g)\sim \mathcal {N}(\log (0.4),\log (2)^2)$, and *β*_*g*_ from $\log (\beta _g)\sim \mathcal {N}(\log (2),\log (2)^2)$. We implemented different sampling strategies for pseudotime points:

- complete sampling: cells are sampled uniformly across the whole trajectory;
- uneven sampling: cells are sampled from a Gaussian mixture density on the trajectory $\sum _{i=1}^ 5\mathcal {N}(\mu _{t_i},\sigma ^2_{t_i})$, where $\mu _{t_i}$ is randomly sampled along the pseudotime axis; $\sigma ^2_{t_i}$ is randomly generated from *U*(0.5, 2.5).

Since sequencing noise does not affect the phenotype, we simulated the phenotypes based on the absolute mean expression *μ*_*ng*_(*t*). When there are *G*_*Y*_ genes that affect the outcome *Y*_*n*_, we have


(12) \begin{eqnarray*}
\mathbb {E}[Y_n]=f\left\lbrace \sum _{g=1}^{G_Y}\left[\sum _t\mu _{ng}(t)\gamma _g(t)\Delta t\right]+\sum _{p=1}^{P_Y}\theta _pZ_{np}+U_n\right\rbrace ,
\end{eqnarray*}

where *f*(·) is the link function, *P*_*Y*_ = 100, *θ*_*p*_ is the horizontal pleiotropic effect from $\mathcal {N}(0.5,0.05)$, and $U_n\sim \mathcal {N}(0,1)$. We specified different forms of effect size γ_*g*_(*t*) in simulations, including

- constant function (Y1): γ_*g*_(*t*) = γ_*g*_;
- cosine function to mimic the cyclical influences of cell cycles (Y2): γ_*g*_(*t*) = γ_*g*_cos (*t*);
- constant function in a time period (Y3): $\gamma _g(t)=\gamma _g\mathbb {I} (t\in \mathcal {T})$, where gene expression only affects the outcome at a pseudotime period $\mathcal {T}$;
- cosine function in time period (Y4): $\gamma _g(t)=\gamma _g\mathbb {I} (t\in \mathcal {T})\cos (t)$;

where γ_*g*_ is sampled from the normal distribution $\mathcal {N}(0.5,0.05)$.

We simulated genotypes of 10 000 SNPs and expression levels of 100 genes in 500 individuals, and sampled 80–140 cells (representing the number of cells of a certain type in the actual scenario) for each individual from a trajectory containing two linearly arranged branches with a total time length of 20. Each gene was regulated by 15–25 SNPs. We used linear models for quantitative outcomes and logistic models for binary outcomes, with 20 out of 100 were specified as causal genes for each phenotype.

### Real data processing

Applying to onek1k (Supplementary Fig. S1) and Roche_MS (Supplementary Fig. S2) datasets, we illustrate the ti-scMR procedure in real-world data (Supplementary Fig. S3). In the Roche_MS application, we used genotypes and 10× sc-RNA sequencing data containing samples of cortical gray matter and white matter from 53 individuals with multiple sclerosis and 29 healthy controls [25]. In the onek1k application, we employed data from 10× sc-RNA sequencing on peripheral blood mononuclear cells and SNP genotyping with Illumina SNP microarray of 982 samples. We performed genotype calling with the raw fluorescence intensity files provided using Illumina GenomeStudio software with the GSA-24v2-0-A1 array and then converted to VCF format.

We conducted quality control on genotypes using PLINK 2.0. We excluded non-SNP and non-biallelic variants, variants with a call rate <0.95, minimum allele frequency (MAF) <0.01, and variants with a Hardy–Weinberg equilibrium (HWE) *P*-value <1e−6. We performed SNP sorting and chromosome splitting using bcftools. The conform-gt program was used to check and harmonize the strands, and ambiguous SNPs were removed. We utilized the Michigan Imputation Server (https://imputationserver.sph.umich.edu/index.html) for genotype imputation [26]. After imputation, we excluded SNPs with a call rate <0.95, MAF <0.05, and HWE *P*-value <1e−6 again [27].

The expression matrix was processed with quality control and log-normalization following the Seurat standard workflow [28]. Batch effects were corrected using Harmony [16]. Cell-type annotations were provided by the original researches, and outliers in each cluster were removed.

We inferred cell trajectories using slingshot [18]. We calculated the temporal cumulative effects of scaled gene expressions using PACE and then performed time-cumulative eQTL mapping. We selected *cis*-eQTLs with *P*-values <.001 (approximately corresponding to the instrument strength *F* > 10) [29], and *trans*-eQTLs with a more strict threshold (adjusted FDR <0.01), as artifacts usually occur more easily in *trans*-eQTL mapping [30]. SNPs after LD stepwise clumping (*r*^2^ < 0.1) were specified as IVs.

We performed Wilcoxon rank sum DE tests to identify candidate genes that influence the outcome. The differentially expressed genes (*P*_adj_ < .05 and |log_2_FC| > 1.0 in the Roche_MS application; *P*_adj_ < .01 and |log_2_FC| > 2.0 in the onek1k application) were selected as candidate genes for subsequent causality testing. We used the 2SRI estimator with Lasso penalty on horizontal pleiotropy of IVs in ti-scMR.

## Results

### Cumulative effects of gene expressions are proxy that improve the power of sc-eQTL mapping.

We first investigated the impact of different sc-eQTL strategies (Fig. 1B), including average eQTL, time-cumulative eQTL, time-interval-aggregate eQTL, dynamic eQTL, and interactive dynamic eQTL (see the “Materials and methods” section). We specified different forms of eQTL effects (constant or time-varying), and implemented different sampling strategies along the trajectory (complete sampling or uneven sampling with a Gaussian mixture density).

**Figure 2.**
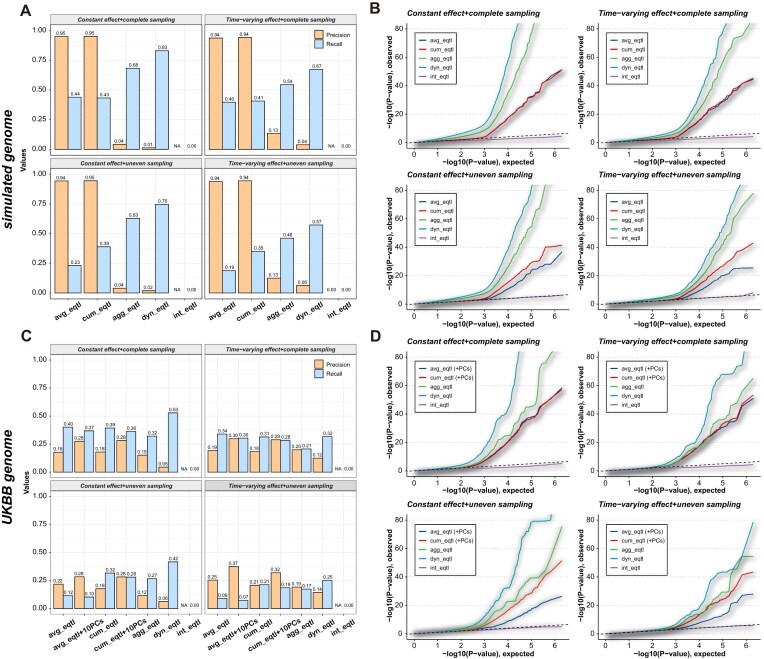
eQTL mappings with different methods in simulations. (**A**) Precision and recall on genuine SNP–gene pair detection in datasets with simulated genotypes of independent loci. (**B**) QQ plot of eQTL mapping in datasets with simulated genotypes of independent loci. (**C**) Precision and recall on genuine SNP–gene pair detection in datasets with real genotypes of loci in LD from UK BioBank. (**D**) QQ plot of eQTL mapping in datasets with real genotypes of loci in LD from UK BioBank. avg_eqtl, average eQTL; cum_eqtl, time-cumulative eQTL; agg_eqtl, time-interval-aggregate eQTL; dyn_eqtl, dynamic eQTL; int_eqtl, interactive dynamic eQTL. NA means no significant SNP–gene pair detected in eQTL mapping.

Although eQTL mapping, as an association test, does not aim to locate causal SNPs; and subsequent MR analysis does not require a causal IV as well, we evaluated the performance on genuine SNP–gene pair detection to conveniently compare the performance of different sc-eQTL mapping strategies. We first simulate genotypes of independent loci, so that we can exclude spurious SNP–gene associations due to LD (Fig. 2A and Supplementary Table S1). We used a threshold of Benjamini–Hochberg (BH) adjusted *P*-values <.05. Here, precision, or true discovery rate (TDR), is defined as the proportion of SNP–gene pairs with true effects among all significant pairs, equal to 1 minus the FDR, and recall, the proportion of significant pairs among all SNP–gene pairs with true effects, represents the test power. When cells are uniformly distributed along the entire trajectory, there is no notable difference between the average and time-cumulative eQTL mapping. However, when cells are unevenly distributed along the trajectory, using the cumulative effect inferred through PACE can improve the power noticeably while controlling the same level of FDR, no matter whether δ_*pg*_(*t*) varies with pseudotimes.

We also employ QQ plots to diagnose the behavior of eQTL mapping. A well-calibrated test should follow the diagonal initially, followed by inflation in tails indicating true signals [31]. A dramatic *P*-inflation can be observed for time-interval-aggregate and dynamic eQTL mappings, resulting in a high risk of false discovery. Testing the interactive effects, on the other hand, leads to serious deflation, resulting in few or even no SNPs detected (Fig. 2B).

When using synthesized phenotypes based on real genotypic data containing SNPs in LD from European ethnic individuals in UK BioBank (Fig. 2C and Supplementary Table S2), the precision will decrease significantly due to LD (noted that precision is defined for causal SNP while eQTL mapping tests marginal association), while the decrease in recall is relatively small. A similar trend can be observed. Time-cumulative eQTLs will achieve a better power if the observed cells of each individual are unevenly distributed on the trajectory. For real genotype data, the introduction of PCs of genotypes as covariates can efficiently reduce FDR for cumulative eQTL. However, for time-interval-aggregate, dynamic and interactive dynamic eQTLs, cells of the same individual are rearranged to different “pseudo-individuals” with the same genotypes, and therefore introducing genomic PCs as covariates contributes to serious collinearity, hindering the estimation. QQ plots (Fig. 2D) support the above findings.

### Temporal information recovered from trajectories contributes to the identification of causal genes through MR

We evaluated the performance of ti-scMR in detecting causal genes using the simulated single-cell transcriptomic dataset based on real genotypic data from UK BioBank, with eQTL effect size varying over time. Cells were sampled unevenly from the trajectory. We used SNPs discovered by time-cumulative eQTL as IVs. Different forms of gene–phenotype effects γ_*g*_(*t*) were simulated, including constant effect at all time (Y1), time-varying effect at all time (Y2), constant effect at a particular period (Y3), and time-varying effect at a particular period (Y4). We repeated 50 times in each scenario and reported the average.

**Figure 3.**
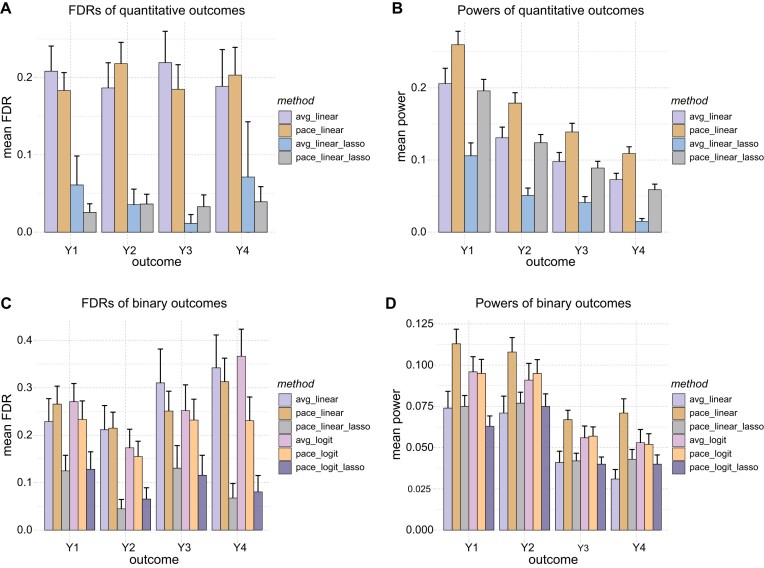
Performance of different MR methods. (**A**) FDRs of different methods on quantitative outcomes. (**B**) Powers of different methods on quantitative outcomes. (**C**) FDRs of different methods on binary outcomes. (**D**) Powers of different methods on binary outcomes. We simulated different modes of gene-trait effects. Y1: the effect of gene expression on outcome is constant along the trajectory; Y2: the effect of gene expression on outcome is time-varying (cosine function of time) along the trajectory; Y3: the effect of gene expression on outcome is constant but only influence in a particular time period; and Y4: the effect of gene expression on outcome is time-varying (cosine function of time) and only influence in a particular time period. In all scenarios, the eQTL (SNP–gene) effects are time-varying and cells are unevenly sampled. We repeat 50 times simulations in each scenario. The bars show the average performance and the error bars show the standard error of the average values. Genius causal genes were randomly selected in simulations. Putative causal genes were determined using criteria BH adjusted *P*_adj_ ≤ .05.

We measured the FDRs and statistical powers of single-cell MR (Fig. 3). For quantitative outcomes (Fig. 3A and B, and Supplementary Table S3), we compared 2SLS estimators using the individual mean (avg_linear), the cumulative expression effect (pace_linear), and the cumulative expression effect with control for horizontal pleiotropy via Lasso penalty (avg_linear_lasso and pace_linear_lasso). Basically, estimators using PACE have a significant improvement in power over the simple average method regardless of the specific forms of γ(*t*). Furthermore, controlling horizontal pleiotropic effects with Lasso can further reduce FDRs.

For hypothesis testing purposes, a binary outcome can be simply treated as a continuous response and fitted with the ordinary 2SLS procedure (avg_linear, pace_linear, pace_linear_lasso) [32]. Additionally, we implemented 2SRI estimators [13, 20, 21] with logistic model (avg_logit, pace_logit, pace_logit_lasso) as well (Fig. 3C and D, and Supplementary Table S4). When using 2SLS, like performance with quantitative outcomes, using the cumulative effects of gene expressions calculated by PACE can achieve an improvement in power. Although the differences in power between simple average and PACE diminish when using logistic models, PACE can reduce FDR of 2SRI estimators. Regulation by Lasso can efficiently control FDR for both 2SLS and 2SRI estimators, while the 2SRI estimator does not show better performance than the 2SLS that simply treats binary outcomes as continuous variables. In general, the introduction of temporal information has improved the statistical power while controlling the FDR.

We further compare ti-scMR (using time-cumulative expression effect in eQTL and MR, plus lasso regularity in MR) with simple DE, GIFT [33] (the version using individual-level data), and vanilla single-cell MR (simply using average expression among cells in eQTL and MR), under the same settings of Fig. 3, i.e. real genomes, time-varying eQTL effects, uneven sampled cells along trajectory, and different kinds of gene-trait effects. For all methods, we use a BH-adjusted *P*-value of <.05 as the detection threshold (Supplementary Fig. S4). Although DE and GIFT identify more genes, this comes at the cost of a high FDR. Compared to vanilla single-cell MR, ti-scMR further reduces the FDR while maintaining similar power.

### ti-scMR identified genes with causal links to multiple sclerosis in oligodendrocytes.

We then demonstrated two real-world applications of ti-scMR to illustrate how it can reveal genes that are causally linked to individual phenotypes (e.g. diseases) and cellular phenotypes (e.g. cell types).

Neuroimmune interaction has attracted considerable attention in recent years, as many immune-related diseases have been found to be associated with dysfunction of the nervous system, and it has also been discovered that some neurological disorders (e.g. Parkinson’s disease [34]) have an immune basis. Multiple sclerosis (MS) is a common autoimmune disease and a disorder of the central nervous system. In MS patients, the immune system attacks the myelin sheath wrapping around nerve fibers, leading to damage to nerve fibers and causing inflammation and degenerative changes in the brain and spinal cord. Oligodendrocytes (OLs) have been reported to be closely related to the neural remyelination process in the nervous system. The demyelination process in MS patients is accompanied by complex immune-mediated attacks on OLs [35].

**Figure 4.**
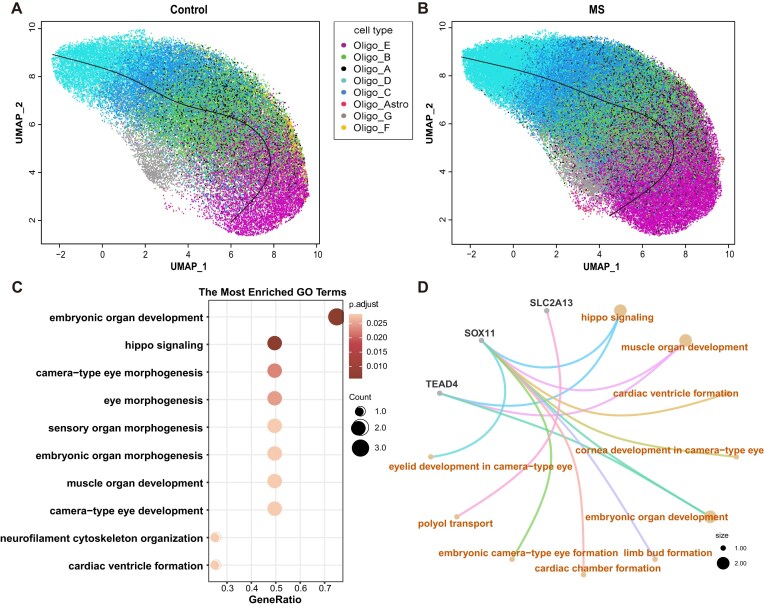
Genes expressed in OLs linked to multiple sclerosis. (**A**) Trajectory of OLs in the control group. (**B**) Trajectory of OLs in the MS group. Labels of OL subtypes are extracted from the original dataset. (**C**) Bubble chart of GO enrichment analysis on gene proportions. (**D**) Network diagram of enriched functions and genes.

Here, we utilized the ti-scMR model to identify the impact of gene transcription changes in OLs on MS. We investigated six potential causal genes in OLs (*P*_adj_ ≤ .1; Supplementary Table S5), which are enriched in several pathways through a subsequent gene ontology (GO) enrichment analysis (Fig. 4). The Hippo pathway is an important mediator in the formation and maintenance of the neural myelin sheath [36]. For example, the *Sox* genes are growth regulators that play roles in sex determination, neural crest development, and neurogenesis, and previous studies have found associations between *Sox* gene expression and MS [37]. *ATP8A2* encodes an ATPase involved in the trans-membrane transport of lipids and plays an important role in physiological activities such as cytoskeleton remodeling and vesicular transport. And *ATP8A2* is reported to be associated with disorders such as intellectual disability, severe hypotonia, choreoathetosis, and optic atrophy [38]. The downregulation of *ATP8A2* expression in OLs of MS patients is believed to be related to methylation regulation and may affect myelin sheath formation by influencing lipid transport [39]. *SLC2A3* encodes the major neuronal glucose transporter 3 (GLUT3), which facilitates glucose transmembrane diffusion and is involved in cellular energy metabolism. GLUT activity is related to various immune activities, including chemokine secretion and targeted cell migration, and is considered a potential target for the treatment of autoimmune diseases [40].

We also compare the results with a gold standard disease gene enrichment dataset [41]. Five gold standard MS-relevant genes are included in our analysis as candidate genes, and two of them show *P*-values <.05 in ti-scMR analysis. This overlap further supports the validity of our method.

### ti-scMR identified genes participating in B cell differentiation

**Figure 5.**
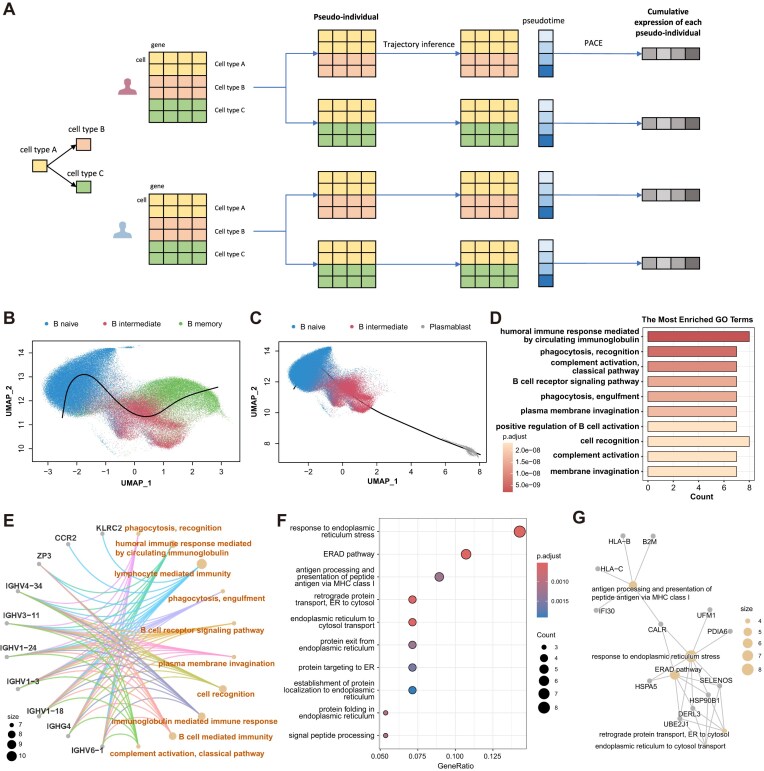
Genes linked to the differentiation of B cells. (**A**) ti-scMR can be extended to cellular phenotypes (e.g. cell type) by recombining cells of different cell types according to differentiation trajectory and then creating “pseudo-individuals.” (**B**) Trajectory of differentiation pathway “naive B cell → intermediate B cell → memory B cell.” (**C**) Trajectory of differentiation pathway “naive B cell → intermediate B cell → plasmablast cell.” (**D**) Histogram of GO enrichment analysis on genes identified by ti-scMR based on candidates pre-filtered via simple DE analysis between memory B cells and plasmablasts. (**E**) Network diagram of enriched functions and genes identified by ti-scMR based on candidates pre-filtered via simple DE analysis between memory B cells and plasmablasts. (**F**) Histogram of GO enrichment analysis on genes identified by ti-scMR based on candidates pre-filtered via tradeSeq. (**G**) Network diagram of enriched functions and genes identified by ti-scMR based on candidates pre-filtered via tradeSeq.

We then extended ti-scMR to cellular phenotypes by rearranging cells of different types and creating new “pseudo-individual” (Fig. 5A).

B cells are the main participants in the humoral immune system. When B cell receptors (BCRs) on the cellular surface specifically recognize homologous antigens, the B cells are activated and differentiate into various antibody-secreting cells (ASCs), including short-lived plasma cells, or, with the assistance of T helper cells in the germinal center (GC), differentiate into plasmablasts and plasma cells. Plasma cells, migrating to the bone marrow, can become long-lived plasma cells. Meanwhile, other GC B cells differentiate into memory B cells, which can survive long term and rapidly differentiate into ASCs upon reencountering the antigen [42].

The selective differentiation process of GC B cells is regulated by various factors, including gene expression regulation, DNA methylation, T–B cell interactions, and cytokines, and has recently received widespread attention and research [43–45]. Here, leveraging the onek1k dataset [46], we utilize the ti-scMR model to examine potential causal genes regulating two distinct differentiation trajectories of GC B cells. The first trajectory represents the path “naive B cell → intermediate B cell → memory B cell” (Fig. 5B), while the second depicts the path from “naive B cell → intermediate B cell → plasmablast cell” (Fig. 5C). To construct these trajectories, the cells from each individual were split and recombined into the two separate paths, effectively generating new “pseudo-individuals” (Fig. 5A).

Thirty-five genes (*P*_adj_ ≤ .01) were identified as potential causal genes that affect B cell differentiation (Supplementary Table S5). GO enrichment analysis shows that these 35 genes are enriched in multiple immune-related pathways, including humoral immune response, immunoglobulin activity, complement function, phagocytosis, BCR signaling, and plasma membrane invagination (Fig. 5). For example, ti-scMR analysis identified several genes associated with immunoglobulin class switching recombination, and V(D)J recombination plays a crucial role in B cell maturation and differentiation [47]. Genes such as *IGHG4* and *IGLV3* have been reported as markers of plasma cells [48]. Chemokine CCR2 participants in the transition from immature B cells to mature B cells [49]. Long noncoding RNAs, such as *IFNG-AS1* have been revealed to be closely associated with processes involving T cells, natural-killing cells, and interferon gamma (IFNγ) [50], a critical driver of GC reactions [42] and B cell polarization [51]. Gene *CES1*, encoding carboxylesterase, is closely associated with energy metabolism in GC B cells, particularly with the transcription factor NF-κB [52].

It is worth noting that some genes encoding those cytokines widely reported to be closely associated with the selection process of GC B cell differentiation [42, 45], such as *PRDM1* (encoding B lymphocyte-induced maturation protein 1 or BLIMP-1) and *IRF4*, were not identified as causal genes due to the absence of suitable eQTLs. Other marker genes such as *XBP1*, *PAX5*, and *BACH2* did not have localized *cis*-eQTLs or their expression levels were not measured in the single-cell dataset as well. This indicates that scMR is limited by the number of donors in sc-RNA datasets, which reduces the number of available SNP markers and results in lower statistical power. Moreover, most eQTLs are specific to cell types and cell states, which exacerbates the difficulty of integrating different differentiation lineages for eQTL mapping for MR analysis with cell types as outcomes.

It is worth noting that, to reduce computational burden, we initially performed DE based on the final differentiation outcomes to preselect candidate genes for ti-scMR. This strategy may have led to the omission of some true causal genes. To address this, we employed an alternative approach using tradeSeq [53], which is specifically designed to identify genes associated with differentiation trajectories. Genes showing significant differences between lineages (*P* < .01) were selected as candidate genes, and ti-scMR was then applied with all other settings unchanged. The results (Fig. 5F and G, and Supplementary Table S6) revealed substantial differences in the set of identified genes. The enriched GO terms predominantly involve ER-associated protein processing pathways, including retrograde transport, ERAD, and unfolded protein response, suggesting that the identified genes may play critical roles in cellular stress responses and antigen presentation mechanisms. It can be seen that, compared to using genes preselected by DE based on final differentiation outcomes, applying ti-scMR to candidate genes identified by tradeSeq—which captures dynamic changes along trajectories—enables the identification of more potential causal genes involved in processes such as cellular stress responses and immune activation during development.

## Discussion

Gene expression has been considered as intermediate links in the causal chain from genotypes to phenotypes. Single-cell DE analysis reveals a large number of genes associated with cellular and individual phenotypes but the causal mechanisms remain unclear due to confounding factors, e.g. complex interactions between genes (epistasis) [54]. As a method leveraging genetic variations as IVs to assess causal relationships, MR has gained widespread attention and application in epidemiology and genetics. Recent studies start to extend MR framework to molecular traits like gene expressions and protein abundance, integrating information from different omics to infer causal pathways. For example, TWAS, which can be seen as a special two-sample MR framework [32], integrates eQTL and GWAS to identify causal genes affecting phenotypes or diseases. However, these approaches in statistical genetics are usually limited to the population level, without considering heterogeneity among various cell types and states, as well as temporal differences along developmental stages.

Recent epidemiological research started to discuss the limitations of traditional MR methods for time-varying exposures [55, 56], and developed adapted methods, such as multivariable Mendelian randomization [57] and *g*-estimation of structural nested mean models [58]. The former assumes that exposure has a causal effect on the outcome only at specific time points, and these time points of exposures need to be measured and correctly specified in the model [57, 59], while the latter requires all samples to be measured at the same time points and assumes that the effects of IVs remain constant during unmeasured time periods [58]. These assumptions are usually tough to satisfy in single-cell scenarios.

In this article, we proposed ti-scMR, integrating single-cell transcriptomics within the MR framework to identify transcriptomic features causally linked to cellular and individual phenotypic differences. Our approach utilizes functional data analysis techniques to capture dynamic features in cell development, which are reconstructed through TI. We then employ sc-eQTL mapping to obtain genetic instruments at the single-cell level, followed by transcriptomic MR analysis. With the increasing availability of single-cell QTL studies across various omics modalities [30], ti-scMR can be naturally extend to investigate the causality of other molecular characteristics, such as methylation levels and protein abundances, to phenotypes, providing a practical tool for integrating multiple single-cell omics [60, 61].

The model still has certain limitations. The effectiveness relies on the accuracy of cell-type annotation and TI. Currently, many TI methods have been proposed [62], each based on different assumptions and potentially yielding divergent or even opposite lineages. Incorporating prior knowledge is crucial to obtain meaningful trajectories. Some dynamic modeling methods have also emerged in recent years, leveraging experiment-based cell perturbation data [63, 64]. However, the inherently disruptive nature of sequencing makes long-term tracking of individual cell developmental processes impractical, making trajectory-based methods a necessary complement.

Moreover, single-cell data exhibit complex noise issues, insufficient correction may capture technical noise, while excessive correction may eliminate genuine biological signals [17]. Both SNPs and gene expressions present challenges in high-dimensional variable selection [65]. Variations in methods and parameters during steps such as normalization, batch effect correction, and feature selection can impact the final analysis results [28]. Additionally, single-cell datasets often suffer from limited sample sizes, particularly in tissues where destructive sampling is involved, such as the brain, resulting in a scarcity of genetic polymorphisms and insufficient statistical power. The development in a large-scale single-cell database, such as the single-cell eQTLGen Consortium [66], holds promise for future causal analyses with larger sample size and greater statistical power.

In summary, through the integration of TI, sc-eQTL, and MR, ti-scMR allows a more comprehensive understanding of the dynamic processes underlying cellular development and its implications for exploring causal relationships between gene expression and phenotypic variations at the single-cell level. Serving as a practical cross-omic causal inference tool, ti-scMR will promote a causal understanding on the genetic basis of complex traits and diseases.

## Supplementary Material

lqaf082_Supplemental_File

## Data Availability

The genotypes and single-cell transcriptomes of the onek1k cohort are available at Gene Expression Omnibus (GEO) with accession number GSE196830 (https://www.ncbi.nlm.nih.gov/geo/query/acc.cgi?acc=GSE196830) and CELLxGENE (https://cellxgene.cziscience.com/collections/dde06e0f-ab3b-46be-96a2-a8082383c4a1). The genotypes and single-cell transcriptomes of the Roche MS dataset are available at European Genome-phenome Archive (EGA) with dataset number EGAD00001009169 (https://ega-archive.org/datasets/EGAD00001009169, application required) and Zenodo (https://zenodo.org/records/8338963). All the codes are available at https://github.com/sjl-sjtu/ti-scMR and https://doi.org/10.5281/zenodo.15545888.
