# Supplementary material for: ti-scMR: trajectory-inference-based dynamic single-cell Mendelian randomization identifies causal genes underlying phenotypic differences
Source: NAR Genom Bioinform. 2025 Jul 4;7(3):lqaf082. doi: 10.1093/nargab/lqaf082 (PMC12231591; doi:10.1093/nargab/lqaf082)
Supplement: lqaf082_Supplemental_File [file lqaf082_supplemental_file.pdf]

Supplementary Materials for "ti-scMR: Trajectory-inference-based dynamic single-cell Mendelian randomization identifies causal genes underlying phenotypic differences"

April 25, 2025

Table S1: Performance of eQTL methods on simulated genotypes of independent loci.

Scenario	Method	Precision	Recall
Constant effect+complete sampling	avg_eqtl	0.9503	0.4393
	cum_eqtl	0.9517	0.4327
	agg_eqtl	0.0421	0.6827
	dyn_eqtl	0.0123	0.8306
	int_eqtl	NA [1]	0.0000
Constant effect+uneven sampling	avg_eqtl	0.9438	0.2311
	cum_eqtl	0.9455	0.3893
	agg_eqtl	0.0403	0.6291
	dyn_eqtl	0.0189	0.7459
	int_eqtl	NA	0.0000
Time-varying effect+complete sampling	avg_eqtl	0.9373	0.3969
	cum_eqtl	0.9421	0.4066
	agg_eqtl	0.1336	0.5444
	dyn_eqtl	0.0382	0.6730
	int_eqtl	NA	0.0000
Time-varying effect+uneven sampling	avg_eqtl	0.9389	0.1883
	cum_eqtl	0.9450	0.3505
	agg_eqtl	0.1252	0.4617
	dyn_eqtl	0.0628	0.5730
	int_eqtl	0.0000	0.0000

[1] NA means no significant SNP mapped in eQTL analysis.

Table S2: Performance of eQTL methods in simulations with real genotypes in LD.

Scenario	Method	without PCs		+ 10 PCs	
		Precision	Recall	Precision	Recall
Constant effect+complete sampling	avg_eqtl	0.1778	0.4015	0.2761	0.3684
	cum_eqtl	0.1798	0.3934	0.2817	0.3628
	agg_eqtl	0.1518	0.3214		
	dyn_eqtl	0.0450	0.5286	Colinearity	
	int_eqtl	NA [1]	0.0000		
Constant effect+uneven sampling	avg_eqtl	0.2179	0.1153	0.2833	0.1031
	cum_eqtl	0.1768	0.3168	0.2825	0.2806
	agg_eqtl	0.1165	0.2668		
	dyn_eqtl	0.0629	0.4168	Colinearity	
	int_eqtl	NA	0.0000		
Time-varying effect+complete sampling	avg_eqtl	0.1928	0.3398	0.2981	0.3036
	cum_eqtl	0.1832	0.3143	0.2891	0.2842
	agg_eqtl	0.1993	0.2071		
	dyn_eqtl	0.1248	0.3163	Colinearity	
	int_eqtl	NA	0.0000		
Time-varying effect+uneven sampling	avg_eqtl	0.2540	0.0893	0.3747	0.0740
	cum_eqtl	0.2080	0.2133	0.3196	0.1862
	agg_eqtl	0.1919	0.1740		
	dyn_eqtl	0.1437	0.2515	Colinearity	
	int_eqtl	NA	0.0000		

[1] NA means no significant SNP mapped in eQTL analysis.

Table S3: FDRs and powers of different methods on quantitative outcomes.

outcome	method	FDR		power	
		mean	se	mean	se
Y1	pace_linear	0.183	0.023	0.260	0.019
	pace_linear_lasso	0.026	0.011	0.196	0.016
	avg_linear	0.208	0.032	0.206	0.021
	avg_linear_lasso	0.061	0.037	0.106	0.018
Y2	pace_linear	0.218	0.027	0.179	0.014
	pace_linear_lasso	0.036	0.012	0.124	0.011
	avg_linear	0.187	0.032	0.131	0.015
	avg_linear_lasso	0.036	0.020	0.051	0.010
Y3	pace_linear	0.185	0.032	0.139	0.012
	pace_linear_lasso	0.033	0.015	0.089	0.009
	avg_linear	0.219	0.041	0.098	0.012
	avg_linear_lasso	0.011	0.011	0.041	0.008
Y4	pace_linear	0.203	0.036	0.109	0.009
	pace_linear_lasso	0.039	0.019	0.059	0.008
	avg_linear	0.189	0.048	0.073	0.009
	avg_linear_lasso	0.071	0.071	0.015	0.004

We simulated different modes of gene-trait effects. Y1: the effect of gene expression on outcome is constant along the trajectory; Y2: the effect of gene expression on outcome is time-varying (cosine function of time) along the trajectory; Y3: the effect of gene expression on outcome is constant but only influence in a particular time period; Y4: the effect of gene expression on outcome is time-varying (cosine function of time) and only influence in a particular time period. In all scenarios, the eQTL (SNP-gene) effects are time-varying and cells are unevenly sampled.

Table S4: FDRs and powers of different methods on binary outcomes.

outcome	method	FDR		power	
		mean	se	mean	se
Y1	pace_linear	0.266	0.038	0.113	0.009
	pace_linear_lasso	0.125	0.033	0.075	0.007
	avg_linear	0.229	0.048	0.074	0.010
	pace_logit	0.233	0.039	0.095	0.008
	pace_logit_lasso	0.128	0.036	0.063	0.006
	avg_logit	0.271	0.038	0.096	0.009
Y2	pace_linear	0.215	0.033	0.108	0.009
	pace_linear_lasso	0.045	0.020	0.077	0.007
	avg_linear	0.212	0.051	0.071	0.010
	pace_logit	0.155	0.032	0.095	0.008
	pace_logit_lasso	0.066	0.024	0.075	0.008
	avg_logit	0.174	0.039	0.091	0.010
Y3	pace_linear	0.251	0.042	0.067	0.006
	pace_linear_lasso	0.131	0.048	0.042	0.005
	avg_linear	0.310	0.071	0.041	0.007
	pace_logit	0.232	0.044	0.057	0.006
	pace_logit_lasso	0.116	0.042	0.040	0.004
	avg_logit	0.252	0.054	0.056	0.007
Y4	pace_linear	0.313	0.049	0.071	0.009
	pace_linear_lasso	0.068	0.030	0.043	0.006
	avg_linear	0.342	0.069	0.031	0.006
	pace_logit	0.231	0.050	0.052	0.006
	pace_logit_lasso	0.081	0.034	0.040	0.006
	avg_logit	0.367	0.057	0.053	0.008

We simulated different modes of gene-trait effects. Y1: the effect of gene expression on outcome is constant along the trajectory; Y2: the effect of gene expression on outcome is time-varying (cosine function of time) along the trajectory; Y3: the effect of gene expression on outcome is constant but only influence in a particular time period; Y4: the effect of gene expression on outcome is time-varying (cosine function of time) and only influence in a particular time period. In all scenarios, the eQTL (SNP-gene) effects are time-varying and cells are unevenly sampled.

Table S5: Causal genes identified by ti-scMR.

ENSEMBL ID	Gene Name	Chr	Start_pos	End_pos	Strand	DE		ti-scMR	
						P	P_adj	P	P_adj
B cell differentiation									
ENSG00000167476	*JSRP1*	19	2252252	2269759	-	0	0	1.00E-16	2.86E-16
ENSG00000167476	*LOC105372240*	-	-	-	NA	0	0	1.00E-16	2.86E-16
ENSG00000281103	*TRG-AS1*	7	38330978	38378804	+	0	0	1.00E-16	2.86E-16
ENSG00000170027	*YWHAG*	7	76326799	76358991	-	0	0	1.00E-16	2.86E-16
ENSG00000255733	*IFNG-AS1*	12	67989445	68234686	+	0	0	1.00E-16	2.86E-16
ENSG00000188372	*ZP3*	7	76397518	76442071	+	0	0	1.00E-16	2.86E-16
ENSG00000197888	*UGT2B17*	4	68537173	68576413	-	0	0	1.00E-16	2.86E-16
ENSG00000169715	*MT1E*	16	56625475	56627112	+	0	0	1.00E-16	2.86E-16
ENSG00000253755	-	-	-	-	NA	6.20E-262	1.24E-258	1.00E-16	2.86E-16
ENSG00000211933	*IGHV6-1*	14	105939756	105940253	-	7.83E-259	1.57E-255	1.00E-16	2.86E-16
ENSG00000198848	*CES1*	16	55802851	55833337	-	4.64E-158	9.28E-155	1.00E-16	2.86E-16
ENSG00000100196	*KDELR3*	22	38468078	38483447	+	1.77E-155	3.53E-152	1.00E-16	2.86E-16
ENSG00000211892	*IGHG4*	14	105620506	105626066	-	9.11E-124	1.82E-120	1.00E-16	2.86E-16
ENSG00000196431	*CRYBA4*	22	26621963	26630669	+	7.91E-117	1.58E-113	1.00E-16	2.86E-16
ENSG00000211647	*IGLV5-48*	22	22352940	22353433	+	1.48E-97	2.95E-94	1.00E-16	2.86E-16
ENSG00000087842	*PIR*	X	15384799	15493564	-	1.83E-66	3.65E-63	1.00E-16	2.86E-16
ENSG00000211945	*IGHV1-18*	14	106184901	106185394	-	1.48E-59	2.96E-56	1.00E-16	2.86E-16
ENSG00000211934	*IGHV1-2*	14	105986582	105987083	-	6.28E-55	1.26E-51	1.00E-16	2.86E-16
ENSG00000239855	*IGKV1-6*	2	88966262	88966767	-	2.79E-52	5.58E-49	1.00E-16	2.86E-16
ENSG00000211962	*IGHV1-46*	14	106511117	106511856	-	2.80E-52	5.61E-49	1.00E-16	2.86E-16
ENSG00000211649	*IGLV7-46*	22	22369614	22370087	+	7.39E-44	1.48E-40	1.00E-16	2.86E-16
ENSG00000211935	*IGHV1-3*	14	106005095	106005574	-	3.23E-43	6.46E-40	1.00E-16	2.86E-16
ENSG00000121552	*CSTA*	3	122325248	122341969	+	6.16E-08	1.23E-04	1.00E-16	2.86E-16
ENSG00000147570	*DNAJC5B*	8	66021553	66101245	+	8.54E-21	1.71E-17	5.00E-04	1.37E-03
ENSG00000121807	*CCR2*	3	46353864	46360940	+	1.65E-199	3.30E-196	2.00E-03	4.34E-03
ENSG00000211950	*IGHV1-24*	14	106276548	106277043	-	1.77E-76	3.54E-73	2.00E-03	4.34E-03
ENSG00000211941	*IGHV3-11*	14	106116635	106117204	-	5.71E-52	1.14E-48	2.00E-03	4.34E-03
ENSG00000228221	*LINC00578*	3	177441910	177767379	+	1.61E-35	3.21E-32	2.00E-03	4.34E-03
ENSG00000236790	*LINC00299*	2	7988683	8488284	-	2.07E-30	4.13E-27	2.00E-03	4.34E-03
ENSG00000205809	*KLRC2*	12	10426854	10442300	-	1.16E-22	2.33E-19	2.00E-03	4.34E-03
ENSG00000211956	*IGHV4-34*	14	106373663	106374145	-	4.55E-78	9.09E-75	3.00E-03	6.10E-03
ENSG00000107317	*PTGDS*	9	136975092	136981742	+	1.61E-30	3.22E-27	3.00E-03	6.10E-03
ENSG00000166428	*PLD4*	14	104924713	104937761	+	1.11E-52	2.22E-49	4.00E-03	7.64E-03
ENSG00000185745	*IFIT1*	10	89392546	89406487	+	2.08E-25	4.16E-22	4.00E-03	7.64E-03
ENSG00000211669	*IGLV3-10*	22	22811747	22812281	+	2.53E-51	5.06E-48	4.50E-03	8.34E-03
MS in oligodendrocytes									
ENSG00000132932	*ATP8A2*	13	25371974	26025851	+	0	0	1.00E-16	8.85E-15
ENSG00000151229	*SLC2A13*	12	39755025	40106089	-	3.29E-23	1.12E-18	1.00E-16	8.85E-15
ENSG00000226137	*BAIAP2-DT*	17	81029130	81034881	-	4.92E-12	1.67E-07	1.00E-16	8.85E-15
ENSG00000283064	-	-	-	-	NA	7.16E-12	2.43E-07	1.00E-16	8.85E-15
ENSG00000197905	*TEAD4*	12	2959330	3040676	+	8.01E-103	2.72E-98	5.00E-04	3.54E-02
ENSG00000176887	*SOX11*	2	5692384	5701385	+	5.49E-121	1.86E-116	1.50E-03	8.85E-02

Table S6: Causal genes identified by ti-scMR in onek1k using candidate genes detected by tradeSeq.

gene	SYMBOL	chromosome_name	start_position	end_position	tradeSEq_p	ti-scMR_p	ti-scMR_padj
ENSG00000211896	IGHG1	14	1.06E+08	1.06E+08	0	0.001	0.001589
ENSG00000184009	ACTG1	17	81509413	81523847	0	0.001	0.001589
ENSG00000166598	HSP90B1	12	1.04E+08	1.04E+08	0	0.001	0.001589
ENSG00000158710	TAGLN2	1	1.6E+08	1.6E+08	0	0.001	0.001589
ENSG00000239264	TXNDC5	6	7881517	7910788	0	0.001	0.001589
ENSG00000166710	B2M	15	44711358	44718851	0	0.001	0.001589
ENSG00000166562	SEC11C	18	59139866	59158832	0	0.001	0.001589
ENSG00000179218	CALR	19	12938578	12944489	0	0.001	0.001589
ENSG00000172183	ISG20	15	88635670	88656483	0	0.001	0.001589
ENSG00000211892	IGHG4	14	1.06E+08	1.06E+08	0	0.001	0.001589
ENSG00000135916	ITM2C	2	2.31E+08	2.31E+08	0	0.001	0.001589
ENSG00000103187	COTL1	16	84565596	84618078	0	0.001	0.001589
ENSG00000198833	UBE2J1	6	89326625	89352722	0	0.001	0.001589
ENSG00000084207	GSTP1	11	67583742	67586656	0	0.001	0.001589
ENSG00000143933	CALM2	2	47160084	47176921	0	0.001	0.001589
ENSG00000074842	MYDGF	19	4641374	4670362	0	0.001	0.001589
ENSG00000099958	DERL3	22	23834503	23839128	0	0.001	0.001589
ENSG00000132432	SEC61G	7	54752250	54759974	0	0.001	0.001589
ENSG00000118363	SPCS2	11	74949261	74979033	0	0.001	0.001589
ENSG00000108518	PFN1	17	4945652	4949061	0	0.001	0.001589
ENSG00000216490	IFI30	19	18173162	18178117	0	0.001	0.001589
ENSG00000089220	PEBP1	12	1.18E+08	1.18E+08	0	0.001	0.001589
ENSG00000143870	PDIA6	2	10783391	10837977	0	0.001	0.001589
ENSG00000172115	CYCS	7	25118656	25125260	0	0.001	0.001589
ENSG00000044574	HSPA5	9	1.25E+08	1.25E+08	0	0.001	0.001589
ENSG00000131871	SELENOS	15	1.01E+08	1.01E+08	0	0.001	0.001589
ENSG00000147649	MTDH	8	97644184	97730260	0	0.001	0.001589
ENSG00000114902	SPCS1	3	52704955	52711148	0	0.001	0.001589
ENSG00000140988	RPS2	16	1962058	1964841	0	0.001	0.001589
ENSG00000124783	SSR1	6	7268306	7347446	0	0.001	0.001589
ENSG00000120686	UFM1	13	38349849	38363619	0	0.001	0.001589
ENSG00000267458	-	-	-	-	1.11E-16	0.001	0.001589
ENSG00000287916	-	-	-	-	1.11E-16	0.001	0.001589
ENSG00000026025	VIM	10	17228241	17237593	4.44E-16	0.001	0.001589
ENSG00000004468	CD38	4	15778275	15853232	2.44E-15	0.001	0.001589
ENSG00000115053	NCL	2	2.31E+08	2.31E+08	7.80E-14	0.001	0.001589
ENSG00000113387	SUB1	5	32531633	32604079	6.78E-12	0.001	0.001589
ENSG00000234745	HLA-B	6	31353872	31367067	1.17E-11	0.001	0.001589
ENSG00000164442	CITED2	6	1.39E+08	1.39E+08	4.53E-11	0.001	0.001589
ENSG00000126709	IFI6	1	27666064	27672212	7.00E-10	0.001	0.001589
ENSG00000125844	RRBP1	20	17613678	17682295	1.03E-09	0.001	0.001589
ENSG00000161960	EIF4A1	17	7572824	7579006	1.56E-09	0.001	0.001589
ENSG00000164032	H2AZ1	4	99948086	99950355	9.75E-09	0.001	0.001589
ENSG00000243678	NME2	17	51165435	51171744	1.31E-08	0.001	0.001589
ENSG00000187837	H1-2	6	26055740	26056470	2.96E-08	0.001	0.001589
ENSG00000204525	HLA-C	6	31268749	31272130	3.64E-08	0.001	0.001589
ENSG00000198668	CALM1	14	90396502	90408268	1.22E-07	0.001	0.001589
ENSG00000079332	SAR1A	10	70147289	70170523	3.64E-07	0.001	0.001589
ENSG00000142227	EMP3	19	48321509	48330553	1.12E-05	0.001	0.001589
ENSG00000100097	LGALS1	22	37675636	37679802	3.80E-05	0.001	0.001589
ENSG00000163041	H3-3A	1	2.26E+08	2.26E+08	4.20E-05	0.001	0.001589
ENSG00000123416	TUBA1B	12	49127782	49131397	8.09E-05	0.001	0.001589
ENSG00000185905	C16orf54	16	29742463	29745990	3.03E-04	0.001	0.001589
ENSG00000120802	TMPO	12	98515579	98550351	4.52E-04	0.001	0.001589
ENSG00000138495	COX17	3	1.2E+08	1.2E+08	0.001035	0.001	0.001589
ENSG00000112081	SRSF3	6	36594353	36605600	0.008745	0.001	0.001589
ENSG00000163584	RPL22L1	3	1.71E+08	1.71E+08	0	0.002	0.003123
ENSG00000253755	-	-	-	-	1.11E-15	0.003	0.004525
ENSG00000179820	MYADM	19	53864763	53876435	8.70E-04	0.003	0.004525
ENSG00000132507	EIF5A	17	7306999	7312463	6.83E-10	0.004	0.005933
ENSG00000157601	MX1	21	41420020	41470071	0.003421	0.005	0.007295

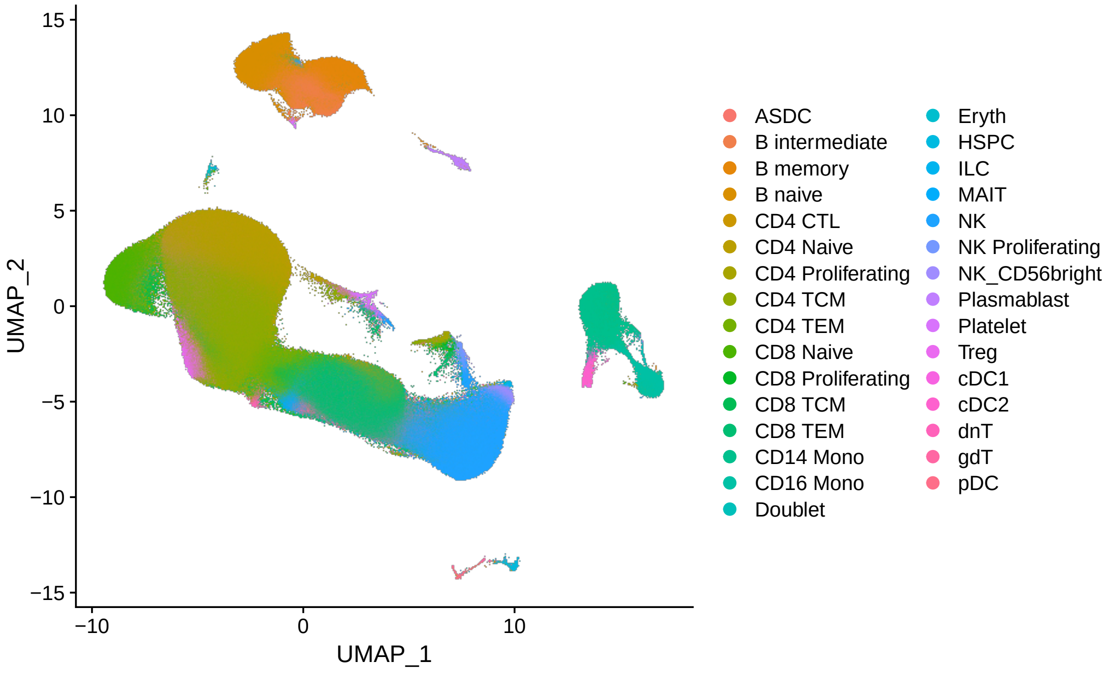

Figure S1: UMAP plot of onek1k single-cell transcriptomic dataset.

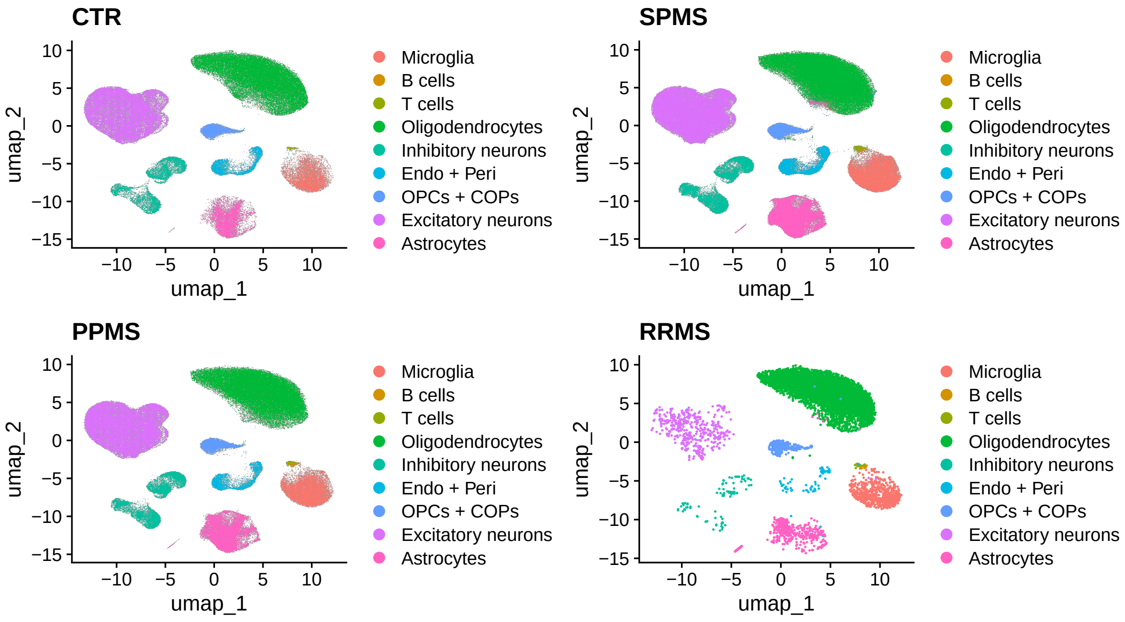

Figure S2: UMAP plot of Roche_MS single-cell transcriptomic datasets.

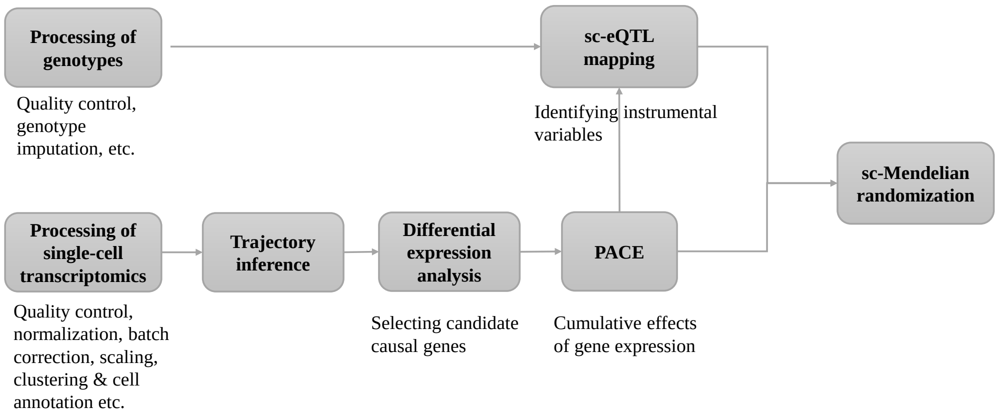

Figure S3: Flowchart of real data processing for ti-scMR.

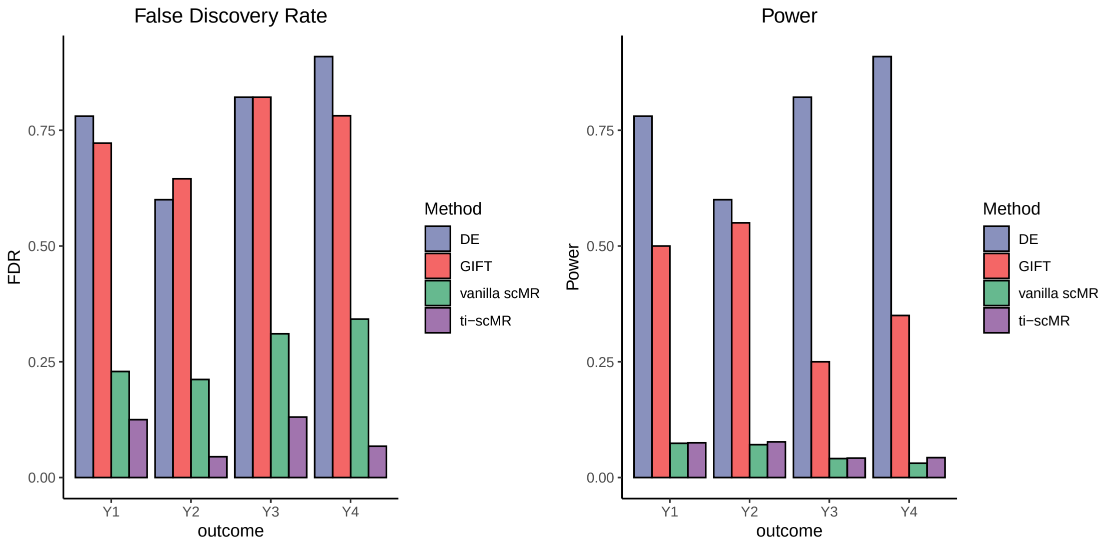

Figure S4: Comparison of ti-scMR with other methods. Simulation settings are the same as the settings in Fig.3, i.e., we use real genomes, time-varying eQTL effects, uneven sampled cells along trajectory, and different kinds of gene-trait effects: constant effect at all time (Y1), time-varying effect at all time (Y2), constant effect at a particular period (Y3), and time-varying effect at a particular period (Y4).
